# Dolphins and sea turtles may host zoonotic parasites and pathogenic bacteria as indicators of anthropic pressure in the Gulf of Taranto (Northern Ionian Sea, Central-Eastern Mediterranean Sea)

**DOI:** 10.1007/s11259-022-10011-y

**Published:** 2022-10-03

**Authors:** Marianna Marangi, Roberto Carlucci, Piero Carlino, Carmelo Fanizza, Gianluca Cirelli, Rosalia Maglietta, Luciano Beneduce

**Affiliations:** 1 Department of the Science of Agriculture, Food, Natural Resources and Engineering (DAFNE), University of Foggia, Napoli 25, 71121 Foggia, Italy; 2 Sea Turtle Research, Rescue and Rehabilitation Center, Natural History Museum of Salento, Calimera, Lecce, Italy; 3 Department of Biology, University of Bari, Bari, Italy; 4 Jonian Dolphin Conservation, Taranto, Italy; 5 Turtle Rescue Centre, Policoro, Italy; 6 Institute of Intelligent Industrial Systems and Technologies for Advanced Manufacturing, National Research Council, Bari, Italy

**Keywords:** Cetaceans, Sea turtles, Sentinel species, Pathogens, Gulf of Taranto

## Abstract

The occurrence of protozoan parasites *Giardia duodenalis* and *Cryptosporidium* spp. such as the pathogenic bacteria *Salmonella* spp. and *Escherichia coli* was molecularly investigated in the following free ranging species of striped dolphins (*Stenella coeruleoalba),* Risso’s dolphins (*Grampus griseus*) as well as loggerhead (*Caretta caretta*) and green (*Chelonia mydas*) sea turtles living in the Gulf of Taranto (Mediterranean Sea). Out of forty-one investigated individuals belonging to the 4 species, 13 (31.7%) were positive to one or more pathogens and zoonotic *G. duodenalis* assemblage A, *Cryptosporidium parvum* and *S. enterica* were identified in striped dolphins*,* loggerhead and green sea turtles*.* In this work, the presence of these opportunistic pathogens has been investigated in fecal samples of free ranging dolphin and sea turtle species for the first time. Moreover, this is the first record of *C. parvum* in loggerhead sea turtles. These results may provide baseline data for the potential role of cetaceans and sea turtles as potential sentinel species for zoonotic and terrestrial pathogens in the marine environment.

## Introduction

The increase in the anthropic pressure in coastal areas and global climate change have raised the emergence of infectious diseases, many of which are zoonotic from agricultural, animal and human waste. Within the concept of the “*one ocean*, *one health”* it is necessary to identify sentinel species that, thanks to their conspicuous nature and capacity, respond to changes in ecosystem structure and function, being able to reflect the health of marine environments (Bossart 2011; de Moura et al. 2014). Among marine vertebrates, cetaceans and sea turtles can have a function as sentinel of marine pollution, due to their long lifespans, global distribution in both coastal and offshore waters, migratory patterns and their ecological role in the marine food web, (Bossart 2011; Pace et al. 2019). Both species can harbor cysts/oocysts of parasites and bacterial pathogens discharged into the coastal waters through sewage, run-off of agricultural, industrial and medical waste (Fayer et al. 2004; Grigar et al. 2017). This condition may not only increase infections and mortality in some populations of marine animals as seals and whales (Aguirre and Tabor 2004; Fayer et al. 2004; Appelbee et al. 2005) but also suggests a fast widespread dispersal of these pathogens of anthropozoonotic origin in marine environments (Plutzer et al. 2018; Reboredo-Fernández et al. 2014, 2015; Srinivas et al. 2020) that may represent a useful biomarker of exposure to polluted waters (Beeby 2001).

The Gulf of Taranto, in the Northern Ionian Sea (Central-Eastern Mediterranean Sea), is characterized by several maritime and land-based human activities. In particular, the area has a noticeable seasonal coastal tourism, a high population density and urbanization of its coastal profile (Ladisa et al. 2010) https://www.agenziapugliapromozione.it/ last access 16/01/2021) and intense ship traffic. In fact, the port of Taranto acts as the endpoint of the Scandinavian-Mediterranean Corridor of the Trans-European Transport Network (TEN-T) (FAO 2014), which it is expected to lead to the further expansion of the logistic platform and intermodal infrastructure to host the forecasted increase in commercial and passenger transport from 2030 (Autorità di Sistema Portuale del Mare Ionio 2017–2030). In addition, the basin is characterized by intense fishing activity (Russo et al. 2017) as well as domestic and industrial discharges that may represent a potential threat to the long-term survival of cetaceans and sea turtle species, due to exposure to pollutants and pathogens (Cardellicchio et al. 2002; Carlucci et al. 2016). On the other hand, the peculiar morphology of the basin characterized by submarine canyons that identify the “Taranto Valley” system and the occurrence of upwelling currents with high seasonal variability (Bakun and Agostini 2001; Capezzuto et al. 2010; Matarrese et al. 2011) contribute to defining the basin as a suitable habitat for several odontocetes and sea turtles.

Recently, the presence of pathogenic bacteria of human and veterinary importance belonging to the *Chlamydiaceae* family has been reported in loggerhead sea turtles from the Mediterranean Sea (Pace et al. 2022)*.* The increasing spread of parasitic and bacterial pathogens from human activity in several marine species and the importance of collecting data, especially in strongly anthropized areas of the Mediterranean Sea, such as the Gulf of Taranto, has encouraged the implementation of this study aimed at providing baseline data for the assessment of the zoonotic infestation of dolphins and sea turtles in coastal areas and their potential role as carriers of zoonotic pathogens, becoming important indicators for public health-related issues (Bossart 2011). This study has used molecular methods to analyze the presence of *Cryptosporidium* spp. and *Giardia duodenalis* as well as *Salmonella* spp. and pathogenic *Escherichia coli* serotypes, in free ranging individuals of striped (*Stenella coeruleoalba*) and Risso’s dolphins (*Grampus griseus*) as well as in loggerhead (*Caretta caretta*) and green (*Chelonia mydas)* sea turtles living in the Gulf of Taranto (Northern Ionian Sea, Central-Eastern Mediterranean Sea).

## Material and methods

### Study area and sampling

**Fig. 1:**
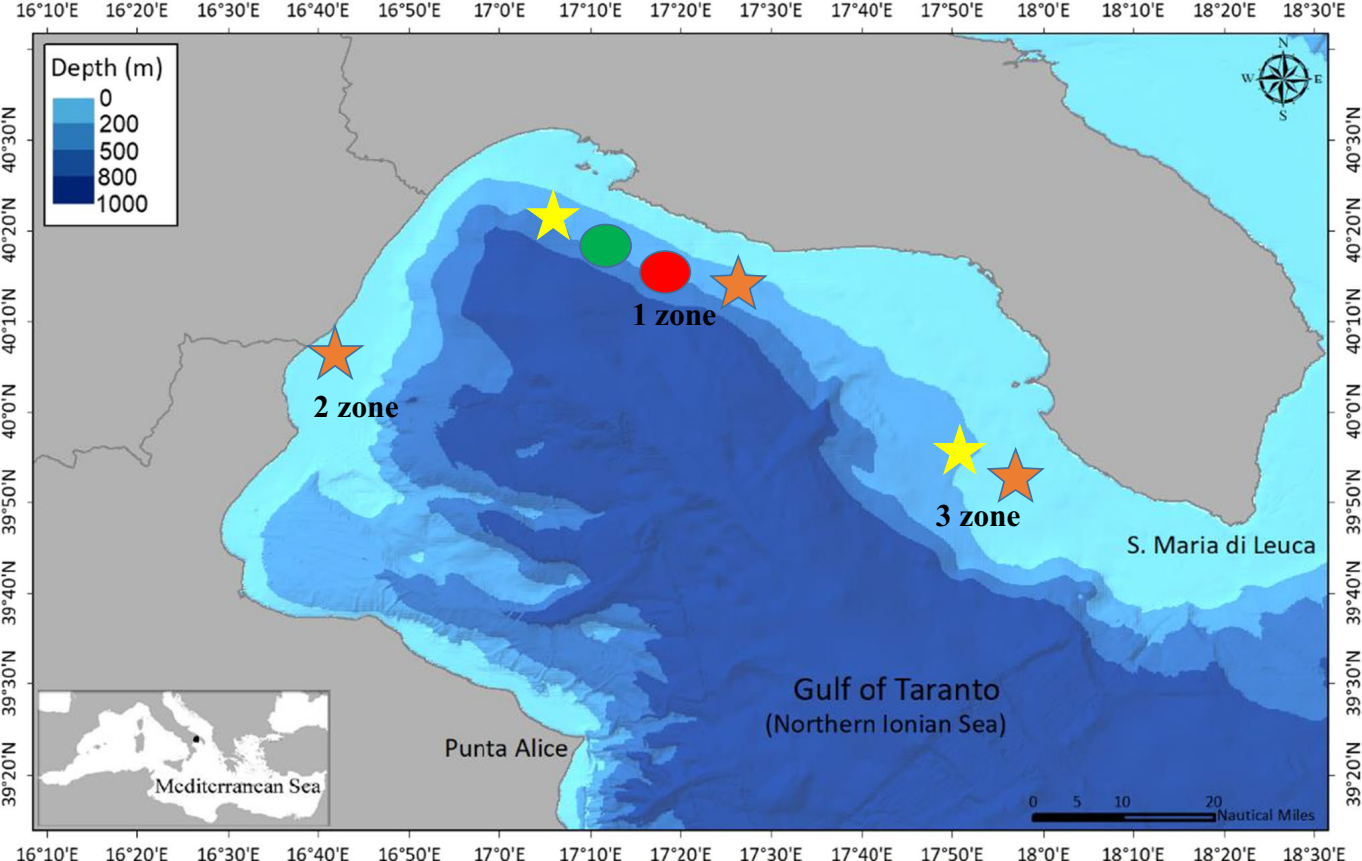
Map of the sampling zones within the Gulf of Taranto and number of dolphin and sea turtle fecal samples collected, *1 zone-TA*: green spot (striped dolphins, no.11); red spot (Risso’ dolphin, no. 7); orange star (loggerhead turtle, no. 5); yellow star (green sea turtle, no. 2), *2 zone-PO*: orange star (loggerhead turtle, no. 8), *3 zone-LE*: orange star (loggerhead turtle, no. 7); yellow star (green sea turtle, no. 1)

The Gulf of Taranto (Fig. 1) hosts several species of cetaceans and sea turtles; in particular, the striped dolphin is the most frequent and abundant species, followed by the common bottlenose dolphin (*Tursiops truncatus*), the Risso’s dolphin and the sperm whale (*Physeter macrocephalus*) (Carlucci et al. 2014; Renò et al. 2019; Azzolin et al. 2020; Carlucci et al. 2020; Maglietta et al. 2020; Papale et al. 2020). Concerning the sea turtles, the most frequent species in the basin is the loggerhead sea turtle, followed by the occasionally seen green and leatherback sea turtles (*Dermochelys coriacea*) (Casale et al. 2018; Pierri et al. 2019).

Since December 2018, one fecal sample was collected for each of 41 free ranging individuals of the four dolphin and sea turtle species within Gulf of Taranto area (Fig. 1 and Table 1).

**Table 1:** Sample ID, time collecting sample, sampling zone, cause of finding of the two dolphin and two sea turtle species circulating within the Gulf of Taranto and involved in the study with the molecular results for the four investigated pathogens, 1 zone: Taranto; 2 zone: Policoro; 3 zone: Lecce; FR: free-ranging; STR: stranded; BCA; By-catch; SD: Striped dolphin; RD: Risso’s dolphin; LT: loggerhead turtle; GT: green sea turtle

					PCRs Pathogens			
Sample ID	Time collecting sample	Sampling zone	Cause of finding	Species	*Giardia duodenalis*	*Cryptosporidium sp.*	*Salmonella sp.*	*Escherichia coli* O157:H7
1	Feb 2019	1 zone	FR	SD	-	-	-	-
2	Feb 2019	1 zone	FR	SD	-	-	-	-
3	Feb 2019	1 zone	FR	SD	Assemblage A	-	-	-
4	Feb 2019	1 zone	FR	SD	-	-	*S. enterica*	-
5	Feb 2019	1 zone	FR	SD	-	-	*S. enterica*	-
6	Mar 2019	1 zone	FR	SD	-	-	-	-
7	Mar 2019	1 zone	FR	SD	-	-	-	-
8	Mar 2019	1 zone	FR	SD	-	-	-	-
9	Mar 2019	1 zone	FR	SD	-	-	-	-
10	Apr 2019	1 zone	FR	SD	Assemblage A	-	-	-
11	Apr 2019	1 zone	FR	SD	-	-	-	-
12	August 2019	1 zone	FR	RD	-	-	-	-
13	August 2019	1 zone	FR	RD	-	-	-	-
14	August 2019	1 zone	FR	RD	-	-	-	-
15	August 2019	1 zone	FR	RD	-	-	-	-
16	August 2019	1 zone	FR	RD	-	-	-	-
17	August 2019	1 zone	FR	RD	-	-	-	-
18	August 2019	1 zone	FR	RD				
19	April 2019	2 zone	STR	LT	-	*C. parvum*	-	-
20	April 2019	1 zone	BCA	LT	-	-	-	-
21	April 2019	1 zone	BCA	LT	-	-	-	-
22	May 2019	1 zone	BCA	LT	-	-	-	-
23	May 2019	1 zone	BCA	LT	-	-	-	-
24	July 2019	2 zone	STR	LT	-	*C. parvum*	-	-
25	August 2019	2 zone	STR	LT	-	-	-	-
26	May 2020	2 zone	BCA	LT	-	-	-	-
27	June 2020	1 zone	BCA	LT	-	-	*S. enterica*	-
28	Sept 2018	3 zone	STR	LT	-	-	-	-
29	Feb 2019	3 zone	STR	LT	-	-	-	-
30	March 2019	2 zone	STR	LT	-	*C. parvum*	-	-
31	June 2019	3 zone	STR	LT	-	-	-	-
32	July 2019	3 zone	STR	LT	-	-	-	-
33	July 2019	2 zone	STR	LT	-	*C. parvum*	-	-
34	Sept 2019	3 zone	BCA	LT	-	-	-	-
35	March 2020	3 zone	STR	LT	-	-	-	-
36	July 2020	2 zone	STR	LT	-	*C. parvum*	-	-
37	July 2020	2 zone	STR	LT	-	C. parvum	-	-
38	Sept 2020	3 zone	STR	LT	-	-	-	-
39	Dec 2018	1 zone	BCA	GT	-	-	*S. enterica*	-
40	June2019	1 zone	STR	GT	-	-	*S. enterica*	-
41	Oct 2019	3 zone	BCA	GT	-	-	-	-

Dolphin fecal samples were collected in the framework of an ecology research project cetaceans; during the winter and summer boat survey, photo identification and floating feces of the individuals were collected, when possible, from free ranging striped (n. 11) and Risso’s (n. 7) dolphins using a fine nylon mesh net, which was changed between each sample, avoiding direct contact with animals as also described in Marangi et al. 2021. Sea turtle fecal samples were collected from 20 loggerhead and 3 green sea turtles found stranded along the Ionian Sea coast, but still alive or caught by fisherman, in three different sampling zones (1, 2 and 3) within the Gulf of Taranto and kept at two Sea Turtle Rescue Centres (Calimera and Policoro), according to the methodology reported in Marangi et al. 2020.

Sample ID, collected species, time of sample collecting, cause of finding and sampling zone are reported in Table 1. The survey data collected, including photo identification, guaranteed that the sampled animals were unique and sampled only once during the monitoring.

All the fecal samples were individually collected in sterile tubes, refrigerated at 5 °C, and delivered to the analysis laboratory within 24 h for future molecular analysis.

### DNA extraction

Genomic DNA was isolated from individual fecal samples using the Qiagen Stool kit (Qiagen, Germany), according to the manufacturer’s instructions. DNA samples were eluted in 50 μL of PCR grade H_2_O, quantified by using a Qubit 2.0 fluorimeter and stored at –20 °C, pending molecular analysis. The individual genomic DNA samples contained approximately from 2 to 100 ng μL^−1^ according to the water content of the fecal sample and dilution with seawater that occurred during sampling. An average concentration of approximately 10 ng for each DNA sample was used for molecular analysis according to the manufacturer’s instructions and amplification protocols.

### *Giardia duodenalis* and* Cryptosporidium* spp. PCR

For the molecular and genetic characterization of *G. duodenalis* and *Cryptosporidium* spp., part of the TPI gene (~ 530 bp) and of GP60 gene (~ 358 bp), were amplified following the nested-PCR protocol as described in Giangaspero et al. 2014.

### *Escherichia coli* and* Salmonella* spp. PCR

The detection of pathogenic bacteria *Salmonella* spp. and *E. coli* O157:H7 were performed by duplex PCR analyses targeting respectively *invA* and *OriC* genes for *Salmonella* (Chiu and Ou 1996; Elizaquível and Aznar 2008) and *Rfb* and *fliC* genes for *E. coli* O157:H7 (Perelle et al. 2004) and as previously reported in Beneduce et al. 2017.

### Amplification and sequencing

All the PCRs were carried out in 25 μL final volume, including 10 μL of Ready Mix REDTaq (Sigma, St. Louis, MO) and 100 pM of each primer. Approximately 10 ng of genomic DNA was incorporated into each reaction and a negative control sample (no-template) and a known positive control for each pathogen were included in each PCR run.

PCR positive samples were run on 1.2% agarose gel, and positive samples were purified with exonuclease I (EXO I) and thermosensitive alkaline phosphatase (FAST AP) (Fermentas, Whaltham, MA, U.S.A.) enzymes, in accordance with the manufacturer’s instructions. The samples found negative to PCRs were tested twice. Moreover, to be sure that the negative samples were not false negatives, a double concentration of DNA genomic was added to the PCRs mix and subjected to amplifications. Moreover, 16 SrRNA universal primers for bacteria were also used to confirm that microbial DNA was of sufficient quality to be amplifiable through PCR (data not shown).

The PCR fragments obtained were directly sequenced in both directions using the ABI PRIMS Big Dye Terminator v. 3.1 Cycle Sequencing Kit (Applied Biosystems, Foster City, CA, U.S.A.) with the same primers as the respective PCR reactions, in accordance with the manufacturer’s instructions. The sequences obtained were determined using an ABI PRISM 3130 Genetic Analyser (Applied Biosystems), chromatograms were inspected using FinchTV (https://digitalworldbiology.com/FinchTV) and primer regions plus bad-quality regions were trimmed. To investigate the species/assemblages, each sequence was compared with the *G. duodenalis*, *Cryptosporidium* spp., *Salmonella* spp. and *E. coli* homologous nucleotide sequences available in the GenBank database using the BLAST program (Basic Local Alignment Search Tool; https://blast.ncbi.nlm.nih.gov).

Subsequently the sequences were aligned using the CLUSTALW implementation of BIOEDIT, version 7.0.5 (http://www.mbio.ncsu.edu/BioEdit/bioedit.html).

### Statistical analyses

A principal component analysis (PCA) with the dataset of the 23 sea turtle samples and 17 dolphin samples collected in the 3 different sampling zones, using the molecular results of the four investigated pathogens and the main environmental variables (time sample collecting, sampling zone, species, cause of finding) was conducted with Past 4.03 software (Hammer et al. 2001). Permanova analysis was also conducted to evaluate the significance of the single group of variables related to the individuals found positive from each of the investigated pathogens.

## Results

Overall, out of forty-one DNA samples subjected to molecular analysis, 13 (31.7%) were found to be positive to PCRs for one or more pathogens and in particular 2/13 (15.4%) were positive to PCR for *G. duodenalis*, 6/13 (46.1%) were positive to PCR for *Cryptosporidium* spp. and 5/13 (38.5%) were positive for *Salmonella* spp. All the samples were negative to PCRs for *E. coli* O157:H7. As regards the investigated animals, *G. duodenalis* was identified in two individuals of striped dolphin, *Cryptosporidium* in six individuals of loggerhead sea turtle and *Salmonella* in two individuals of striped dolphin and three of sea turtles, of which 1 loggerhead and 2 green sea turtles. Risso’s dolphins were negative for all the investigated pathogens. All the molecular results are reported in Table 1.

After sequencing, *G. duodenalis* assemblage A, *C. parvum* and *S. enterica* were identified and confirmed for DNA homology.

**Fig. 2:**
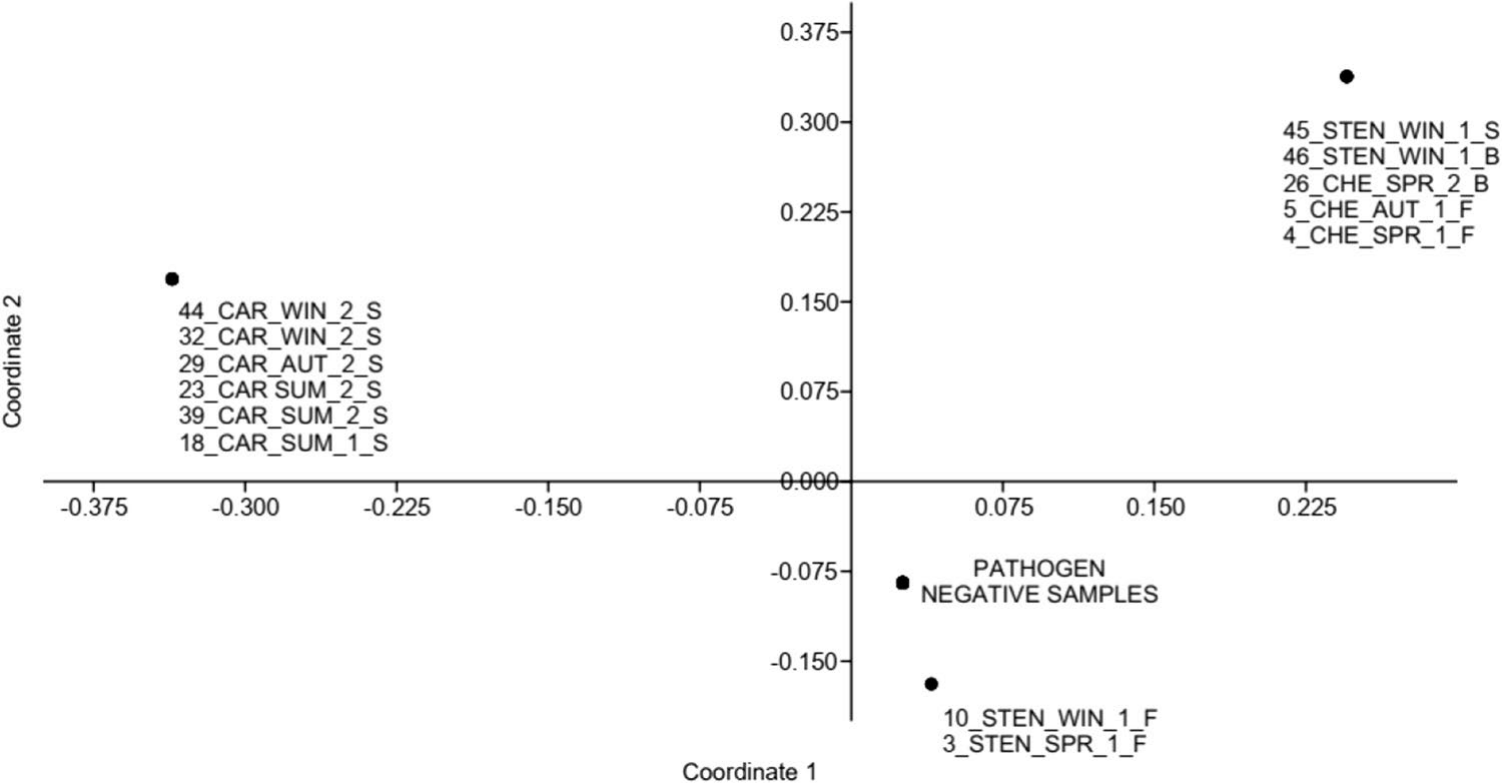
Principal Component Analysis (PCA) on pathogen distribution, CAR = *Caretta caretta*, CHE = *Chelonia mydas,* STEN = *Stenella coeruleoalba;* S = *stranded,* B = *bycatch,* F = *free ranging;* 1–2-3 = *sampling zone*

PCA analysis showed that the main variables that statistically explain the positive pathogen samples are differently correlated between bacterial (*Salmonella*) and parasitic (*Cryptosporidium, Giardia*) pathogens. In fact, *Cryptosporidium* positive turtles were uniquely related to zone 2 stranded *C. caretta*, while *Salmonella* positive turtles were found uniquely within zone 1 and were in all cases bycatch turtles belonging to both sampled turtle species (Fig. 2).

The Permanova test was used as a more robust statistical evaluation of the correlation between pathogen positive samples and environmental variables: as can be seen in Table 2, significant differences were found between *Salmonella* positive samples and marine species, since *G. griseus* was the only marine species that was never found positive to this pathogen. As for location of finding and cause of finding, the Permanova analysis was only significant for the pathogen *Cryptosporidium*, due to the fact that it was found only in individuals from the zone 2 and in the case of stranded animals.

**Table 2:** Permanova analysis (based on Bray–Curtis distance) conducted on the main sampling variables, The statistic was considered significant for *p* < 0,05. The numbers with a statistical evidence are reported in bold

Pathogens	PERMANOVA (*P value*)			
	Time collecting sample	Species	Sampling zone	Cause of finding
*Cryptosporidium*	0,9678	0,1043	0,0277*	0,0006*
*Giardia*	0,6023	0,2259	0,5532	0,7402
*Salmonella*	0,4313	0,0213*	0,3792	0.3292

## Discussion

This is the first environmental study investigating the presence of parasites and bacteria in free ranging species of dolphins and sea turtles, stranded but still alive or caught by fisherman in the Gulf of Taranto. The zoonotic *G. duodenalis* assemblage A, *C. parvum* and *S. enterica* were found in striped dolphins and in two species of sea turtles, namely loggerhead and green turtles.

*G. duodenalis* and *Cryptosporidium* spp. are emerging water- and foodborne enteric zoonotic pathogens (Xiao and Feng 2017) able to infect humans and a wide range of animals, both domestic and wild (Santin 2020). *G. duodenalis* cysts and *Cryptosporidium* oocysts may be released into the terrestrial and marine environment through human/animal excreta (Santin 2020). Cetaceans and sea turtles may become infected either via contamination of coastal waters by sewage, run-off and agricultural and medical waste or by consumption of infected prey such as fish and shellfish, resulting in increased infections and mortality in some populations (Fayer et al. 2004).

In recent studies, *G. duodenalis* and *Cryptosporidium* spp. infections, including zoonotic assemblages (assemblage A) and species (*C. parvum*), are reported in several cetacean species such as bowhead whale (*Balaena mysticetus),* North Atlantic right whale (*Eubalaena glacialis*), harbor porpoise *(Phocoena phocoena),* minke whale *(Balaenoptera acutorostrata),* common bottlenose dolphin, striped and short-beaked common dolphins (*Delphinus delphis*) from the European Atlantic Sea (Reboredo-Fernández et al. 2014, 2015; Hughes-Hanks et al. 2005; Grilo et al. 2018). Within the Mediterranean Sea, *G. duodenalis* and *Cryptosporidium* spp. infections have been reported for Indo-Pacific bottlenose dolphins (*Tursiops aduncus*) and sperm whales (Kleinertz et al. 2014; Hermosilla et al. 2018).

In this study three striped dolphins were found to be positive to *G. duodenalis* assemblage A, and this finding is in line with reports in striped dolphins stranded along the European Atlantic Coast (Reboredo-Fernández et al. 2015). Therefore, to the best of our knowledge, this is the first report of a zoonotic assemblage detected in a dolphin species living within the Mediterranean Sea.

Moreover, six loggerhead sea turtles were found to be positive to *C. parvum*. To date, no data about the presence of *Giardia* and/or *Cryptosporidium* cysts/oocysts in any sea turtles are available. Oocysts of *Cryptosporidium* and zoonotic species (*C. parvum*) have been reported in several species of tortoises (Traversa et al. 2008). Therefore, at the time of writing, this is the first report of *C. parvum* in *C. caretta* and this result extends the known host range of this zoonotic protozoan. However, the possible role of sea turtles as a reservoir of *C. parvum* and also a biomarker indicator for water contamination needs to be further investigated.

The positivity of striped dolphins and loggerhead turtles to *Giardia* and *Cryptosporidium* provides several points of discussion; although the source of infection by these protozoan parasites could not be assessed, different considerations and hypotheses can be drawn about *Giardia* and *Cryptosporidium* contamination in the Gulf of Taranto marine waters and the transmission pathways in these marine species.

The area surrounding the Gulf of Taranto coastal zone is characterized by intense human activities in the form of industries, livestock production and urbanization (Ladisa et al. 2010). Therefore, it can be assumed that runoff from urban, and rural landscapes, and wastewater outfalls can carry *Giardia* and *Cryptosporidium* cysts/oocysts into the coastal water, contributing to a rapid and widespread dispersal, especially after rainfall. Moreover, the large quantities of wastewater discharged from human, animal and industrial sources may contribute to contaminating shellfish and infecting many species of marine animals.

Cetaceans and sea turtles can be parasitized by a rich variety of endoparasites (George 1997). However, it must be highlighted that the differences in the parasite communities of marine species are mainly ascribed to ecological and ontogenetic factors (e.g. trophic conditions, deep/shallow waters, pelagic/benthic diet, food intake rate, type of food). For example, focusing on the feeding habits of sea turtles, the loggerhead feeds primarily on gelatinous plankton (jellyfish and tunicates) and crustaceans and molluscs, with fish and squid as supplementary items (Ladisa et al. 2010) whereas green sea turtles are thought to be largely herbivorous at most life history stages (Bjorndal 1997) even if during the post-pelagic stage this species is likely to be omnivorous (Godley et al. 1998), foraging on a variety of resources including animal prey (Lazar et al. 2010; Carrion-Cortez et al. 2010). The differences in diet composition of sea turtle species could explain the positivity of loggerheads to *Cryptosporidium* and the negativity of green sea turtles to the same parasite.

In the same way the ecological issues and its spatial distribution of Risso’s dolphin on off-shore grounds relatively far from the source of such kind of microbial contaminations could explain negativity to both parasites for Risso’dolphins as compared with the positivity to *Giardia* for striped dolphins.

The positivity of dolphins and sea turtles to *Giardia* and *Cryptosporidium* raise concern for these animals’ health in coastal waters as well for humans who eat raw shellfish and swim in these waters. Indeed, infected humans or animals often excrete large numbers of these encysted protozoa, and it is known that a very low dose of few cysts is required to initiate an infection (Cacciò 2004).

The genus *Salmonella* includes two species, *Salmonella enterica* and *Salmonella bongori.* The first one is largely restricted to cold-blooded animals and is regarded as a rare opportunist in humans and the second one is further divided into subspecies (I–VI) and serotypes (serovars) based on biochemical, antigenic and serological characteristics. To date, there are over 2500 serotypes described with *S. enterica* subspecies I, including almost all the serotypes known to be pathogenic in humans. Well-recognised pathogens in subspecies I include *S.*ser. *typhi* and *paratyphi,* *S.* ser. *typhimurium* and *S.* ser. *enteritidis*.

*S. typhi* and *S. paratyphi* have no natural hosts other than humans, whilst other serotypes (*S. typhimurium* and *S. enteritidis*) have a range of animal hosts yet also cause human infection.

In our study, two striped dolphins and three sea turtles (one loggerhead and two green) were found to be positive to *S. enterica*. Although many marine species are known to harbor *S. enterica,* the role of the environmental factors affecting *Salmonella* spp. persistence in the marine environment remains poorly understood, together with the transmission pathways to marine organisms. Indeed, this fecal bacterium is not indigenous to the marine environment, and its presence in coastal waters has been linked to heavy rain and storm-generated flows, transporting the contamination from sources to the sea via river waters (Martinez-Urtaza et al. 2004), and to *in situ* defecation by infected marine animals (Davidson et al. 2015). Enteric bacteria may concentrate in sediments as well as in invertebrates and vertebrates of marine environments contaminated with fecal materials for prolonged periods. The presence of zooplankton and suspended particles colonized by *Salmonella* has also been reported, suggesting additional pathways for bacterial dissemination in marine habitats (Miller et al. 2010).

Reports of these pathogens in free ranging dolphins and sea turtles are scarce, and mainly referred to stranded and dead animals. Very recently *S. typhimurium* (*Salmonella* 1,4, [5],12:i was found in three stranded striped dolphins suggesting a potential pathogenic role in this species (Grattarola et al. 2019). Despite turtles being reported as a possible reservoir of *Salmonella,* the route of transmission of this pathogenic species and the pathogenicity for turtles are still not clear (Orós et al. 2020). In a recent survey that took into consideration turtles, eggs, nest sand and marine water (Alduina et al. 2020) no *Salmonella* spp. was found and the same results arose from a report on a stranded turtle in Tuscany (Fichi et al. 2016). Moreover, *Salmonella* was rarely isolated when examining several bacterial species rear seawaters in rearing tanks (Chuen-Im et al. 2019). These findings support the hypothesis that *Salmonella* spp. is not common in marine turtle habitats and can be transmitted only when the habitat is heavily polluted by wastes in the coastal environment.

The different distribution of parasitic and bacterial pathogens in three different zones highlights the possibility that polluted coastal environments may cause transmission of specific pathogens related to the different level and type of pollution. Therefore, it is noteworthy that our study provides evidence that turtles and cetaceans can both host zoonotic and human pathogens non-sporadically, and as a consequence may act as bio-sentinels, with a potential involvement in the transmission of these pathogens in the environment. Despite the relatively low number of samples analyzed, these results are interesting also considering the lack similar studies in the species surveyed for the area under analysis. Further investigation will address the causality of higher pathogens prevalence in specific zones and whether some geographical zones may represent potential hotspots for microbial contamination.

The potential risk to public health of *Giardia, Cryptosporidium* and *Salmonella* circulating in the marine environment should not be neglected, considering the release of pathogens by both carriers and infected live marine species, and the implications for humans sharing the same habitat or working with these species.

The present work focused on molecular tools (PCR, sequencing) to investigate the presence/absence of these pathogens, and to evaluate the type and level of infectious diseases in marine wildlife due to anthropozoonotic pathogens. Based on the obtained results, further research could benefit from a culture-based approach, along with the use of quantitative PCR, investigating the occurrence of these pathogens in marine species. The results obtained in this study suggest a non-sporadic transfer of terrestrial zoonotic and human pathogens from the coastal environment to free ranging species in the Gulf of Taranto. Our findings provide the basis for future studies of the potential role of cetaceans as hosts for zoonotic and terrestrial pathogens in the marine environment and strategies to minimize the potential risk for both wildlife and public health.

## Data Availability

The datasets generated during and/or analysed during the current study are available from the corresponding author on reasonable request.
